# Differential expression of programmed death 1 (PD-1) on various immune cells and its role in human leprosy

**DOI:** 10.3389/fimmu.2023.1138145

**Published:** 2023-04-21

**Authors:** Mohammad Tarique, Huma Naz, Mohd Suhail, Ali Turan, Chaman Saini, Naoshad Muhammad, Hari Shankar, Torki A. Zughaibi, Tabish H. Khan, Neena Khanna, Alpana Sharma

**Affiliations:** ^1^ Department of Biochemistry, All India Institute of Medical Sciences (AIIMS), New Delhi, India; ^2^ Centre for Interdisciplinary Research in Basic Sciences, Jamia Millia Islamia, New Delhi, India; ^3^ King Fahd Medical Research Center, King Abdulaziz University, Jeddah, Saudi Arabia; ^4^ Department of Medical Laboratory Sciences, Faculty of Applied Medical Sciences, King Abdulaziz University, Jeddah, Saudi Arabia; ^5^ Department of Molecular Microbiology and Immunology, University of Missouri, Columbia, MO, United States; ^6^ Department of Radiation Oncology, Washington University in Saint Louis, Saint Louis, MO, United States; ^7^ Department of Pathology and Immunology, Washington University in Saint Louis, Saint Louis, MO, United States; ^8^ Department of Dermatology, All India Institute of Medical Sciences (AIIMS), New Delhi, India

**Keywords:** leprosy, T cells, Tregs, PD-1, IL-10, T cell anergy

## Abstract

Leprosy is a chronic bacterial disease caused by *Mycobacterium leprae*. Leprosy patients have been found to have defects in T cells activation, which is critical to the clearance of the bacilli. Treg cell suppression is mediated by inhibitory cytokines such as IL10, IL-35 and TGF-β and its frequency is higher in leprosy patients. Activation and overexpression of programmed death 1 (PD-1) receptor is considered to one of the pathways to inhibit T-cell response in human leprosy. In the current study we address the effect of PD-1 on Tregs function and its immuno-suppressive function in leprosy patients. Flow cytometry was used to evaluate the expression of PD-1 and its ligands on various immune cells T cells, B cells, Tregs and monocytes. We observed higher expression of PD-1 on Tregs is associated with lower production of IL-10 in leprosy patients. PD-1 ligands on T cells, B cells, Tregs and monocytes found to be higher in the leprosy patients as compared to healthy controls. Furthermore, *in vitro* blocking of PD-1 restores the Tregs mediated suppression of Teff and increase secretion of immunosuppressive cytokine IL-10. Moreover, overexpression of PD-1 positively correlates with disease severity as well as Bacteriological Index (BI) among leprosy patients. Collectively, our data suggested that PD-1 overexpression on various immune cells is associated with disease severity in human leprosy. Manipulation and inhibition of PD-1 signaling pathway on Tregs alter and restore the Treg cell suppression activity in leprosy patients.

## 1 Introduction

*Mycobacterium leprae* causes leprosy, a chronic bacterial disease that mostly affects macrophages and Schwann cells. A clinical manifestation associated with different levels of immune response to *M. leprae* makes it a good model for investigating host-pathogen interactions and immunological dysregulation in humans (1, 2). There are five clinical forms of leprosy, including the tuberculoid pole (TT), which is characterized by a Th1 immune response, a high level of cell mediated immunity, and a high degree of resistance to the pathogen. In the lepromatous (LL) pole, the infection is associated with a Th2 immune response, failure of the cell-mediated immune response, foamy macrophages in the dermis as the result of a large number of bacilli, as well as lesions throughout the body. There are three immunologically unstable forms of leprosy in between these two types, borderline tuberculoid (BT), borderline-borderline (BB), and borderline lepromatous (BL) leprosy, exhibiting traits that oscillate between the two types (3–6). Our group had previously observed that the progression of leprosy (from tuberculoid to lepromatous leprosy) was associated with a Th3 type immune response (7). Moreover, it was also observed that IL-35-producing Tregs were more prevalent in the BL/LL pole of leprosy (8), and that the plasticity of the Tregs was also changed when they were treated with IL-12 and IL-23 (9). Our group has also reported recently that another immunosuppressive group, the gamma delta T-cells, are increased in leprosy patients (10, 11) and that leprosy is associated with an impaired function of T cells to respond against *M. leprae* antigens (12).

It is not possible to completely explain the state of polarized immunity by merely examining the generation of Th1/Th2-like effector cells alone. It has been established that there are other subsets of B and T cells that play important roles in determining the immunity of the host like Tregs, Bregs (12–14) and Th17 cells (15). An important component of the immune system is the T regulatory cells, which, in addition to their role in the maintenance of self-tolerance, also play a key role in a wide range of clinical conditions such as cancer, transplant rejection reactions, and autoimmune diseases (16, 17). Treg cell suppression is mediated by inhibitory cytokines such as IL10, IL-35 and TGF-β (8, 18). A higher number of Tregs were observed in leprosy patients, who expressed increased levels of IL-10 and CTLA-4 but not TGF-β. The proliferation of bacilli in uncontrolled patients suggests that these cells might be pathogenic (19). According to recent research, FoxP3+ induced Tregs may down regulate T cells to cause antigen-specific anergy associated with lepromatous leprosy (12). We previously demonstrated that IL-10 producing Bregs influence T cell fate in a functionally distinct way in leprosy patients. The presence of Bregs enhances the activity of Treg cells, which eventually aids in the pathogenesis of leprosy. We have previously demonstrated that interleukin 10 inhibits T-cell proliferation in leprosy patients through a mechanism in which the immune system is suppressed (14). There is evidence that the programmed death 1 (PD-1)–PD ligand (PD-L) pathway is involved in infection, autoimmune disease, and transplant rejection (20). PD-1 pathway plays a critical role in chronic infections in which pathogens evade host immunity (21). When parasites are infected, PD-L1 expression is increased on macrophages, resulting in anergy in T cells. When *Mycobacterium leprae* infections occur, the proliferation of T-cells is slowed down and cytokine production is altered (22), as well as the function of other immune cells is affected. The blocking of PD-1 led to a significant increase in IFN-γ and IL-17 production by T cells. (12, 23). There is an increased expression of PD-1 and PD-L1 on CD4+, B cells, and CD11c+ cells in leprosy patients (24). Although PD-1/PD-L1 signaling pathway has been identified on Foxp3+ Tregs, its role in regulating the function and the activity of these cells has yet to be fully understood yet, it remains unclear exactly how the PD-1 pathway functions and how it dampens the host effector Tregs cell response in human leprosy. In this context, we aimed to study whether the co-inhibitory molecules PD-1, PD-L1 affect Tregs function, and to determine if novel interventions affect leprosy pathogenesis.

In the current study, the proportion of FoxP3+ Tregs and the expression of PD-1 and its ligands have been analyzed on various immune cells. Moreover, we evaluated the role of PD-1 in Tregs mediated suppression of T effector cells using of PD-1 blockade on FoxP3+ Tregs *in vitro*. We also studied the correlation with PD-1 expression with bacteriological index (BI).

## 2 Materials and methods

### 2.1 Patients, healthy controls and ethics

This study included 40 patients newly diagnosed with leprosy, 20 tuberculoid and 20 lepromatous patients from the Department of Dermatology, All India Institute of Medical Sciences (AIIMS), New Delhi, India. This study did not include patients younger than 18 years of age, those with tuberculosis, pregnant women, those with HIV, those with disease, or those on MDT. The Ridley-Jopling classification was used to determine leprosy patients’ clinical and histological characteristics. A total of 10 healthy volunteers were recruited after written consent was obtained for the addition age match (**Table 1**). This study has been approved ethically by the Institute Ethics Committee, AIIMS in New Delhi, India (IESC/T-417/01.11.2013). Prior to the collection of blood samples, written consent was obtained from the patients.

**Table 1:** Clinical details of 40 newly diagnosed untreated leprosy patients and 10 healthy control subjects.

Clinical types	Number of Patients	Sex		Age	BI	Duration of Disease
		M	F	(years)		
Tuberculoid Leprosy (BT/TT)	20	11	09	20-52	0-0.8	0.5-1.7 Yrs.
Lepromatous Leprosy (BL/LL)	20	10	10	23-51	4.1-6	0.6-2.1 Yrs.
Healthy controls (HCs)	10	05	05	21-54	–	–

Patients were typed based on Ridley Jopling classification, BI; Bacillary Index (mean of six lesional sites) and skin lesions. M; male, F; female. BT: Borderline Tuberculoid, BL: Borderline Lepromatous, LL: Lepromatous Leprosy, HC: Healthy controls.

### 2.2 Isolation and culture of human peripheral blood mononuclear cells

We layered blood of leprosy patients and healthy controls on ficoll-hypaque (Sigma Aldrich, USA) and centrifuged the samples at 300g for 15 minutes to isolate mononuclear cells. Two washes were performed in sterile PBS by centrifuging the cells for 10 minutes at 1,200 rpm. We resuspended washed cells in RPMI 1640 with 10% fetal calf serum (Gibco, CA, USA) and used a hemocytometer to measure cell viability and enumeration. 1 × 106 cells/ml were stimulated with *M. leprae* sonicated antigen (10 µg/ml) kindly provided by P. J. Brennan of Colorado State University. The cultures were stimulated with IL-2 and anti-CD3/CD28 and incubated in 5% CO2 at 37°C for 72 hours after stimulation. Cells were harvested, stained by flow cytometry, and then processed for further analysis.

### 2.3 Flow cytometer staining

Cells were harvested after 72 hours and stained with surface antibodies PE-Mouse Anti-Human CD3 (Clone: SK7), Alexa Fluor 488 Mouse Anti-Human CD4 (RPA-T4), APC-Cy7 Mouse Anti-Human CD25 (Clone: M-A251), APC Mouse Anti-Human CD279 (PD-1) (Clone: EH12.1), PE-Cyanine7 Anti-Human CD274 [programmed death-ligand 1 (PD-L1), B7-H1] (Clone: MIH1), PE-Mouse Anti-Human CD14 (Clone: MφP-9), APC Mouse Anti-Human CD19 (Clone: HIB19), PE-CF594 Mouse Anti-Human CD62L (Clone: DREG-56) and incubated for 30 min at 4°C in the dark. Following surface staining of the cells, the cells were incubated for 15 minutes at room temperature with FoxP3 staining buffer; they were then washed twice and permeabilized for 30 minutes at room temperature with permeabilization buffer (1X) (eBioscience). After two washes and resuspended in staining buffer, cells were incubated with PE Rat Anti-Human IL-10 (Clone: JES3-19F1) and PerCP-Cy5.5 Mouse Anti-Human FoxP3 (Clone: 236A/E7). The staining was done according to the manufacturer’s specifications. Antibodies were purchased from BD Biosciences in San Diego, CA. The cells were fixed in 400 µl of 2% paraformaldehyde and stored at 4°C. A Protein Transport Inhibitor containing Monensin (BD Golgi Stop) was used to inhibit the secretion of cytokines 4h prior to harvesting to facilitate intracellular staining. Data was collected on a FACS Canto flow cytometer (BD Biosciences) and analyzed with FCS Express.

### 2.4 RNA extraction and real-time PCR

We extracted total RNA using the TRIzol reagent (Invitrogen, Carlsbad, CA, USA), as recommended by the manufacturer. RNA quality and concentration were measured using UV spectrophotometers. To design primers, we used the NCBI website www.ncbi.nlm.nih.gov/gene. For cDNA synthesis, 1 µg total RNA was transcribed with cDNA Synthesis Kits (Thermo Scientific, USA). A triplicate cDNA sample was amplified for each group in 384 well plates containing primers for the PD-1 gene and the housekeeping gene GAPDH in the Roche LightCycler^®^ 480 system. Analysis was conducted by normalizing threshold cycle values and expressing them as DCt: mean Ct of gene of interest − mean Ct.

### 2.5 Suppression assay

PD-1+CD25^high^CD4+ T cells (Treg cells) were sorted from PBMCs of tuberculoid and lepromatous leprosy patients using FACS Aria Fusion (BD Biosciences). 1x10^5^ CFSE-labeled (2.5 μM) responder CD8+ T cells from PBMCs of healthy controls were co-cultured with/without PD-1+ Treg cells derived from leprosy patients in the presence of 1x10^6^ irradiated APCs and 0.5 ug/ml anti-CD3 (OKT3) mAb. To block PD-1, anti-PD-1 monoclonal antibodies (nivolumab) or control monoclonal antibodies of a matched isotype (15 µg/ml) were added. Flow cytometry was used to measure cell proliferation 5 days later by dilution of CFSE-labeled cells.

### 2.6 ELISA

The IL-10 cytokine was measured as per manufacturer’s instructions using an ELISA kit (Thermo Fisher Scientific, USA) in the culture supernatant. We tested culture supernatant in duplicate in 96-well plates coated with biotin conjugated anti-human antibodies IL-10 (Nunc, Rochester, NY, USA). The manufacturer’s instructions were followed in order to properly carry out the protocol. An optical density measurement was performed at a wavelength of 450 nm for each well.

### 2.7 Statistical analysis

Differences among groups were evaluated using one way ANOVA and Student’s t-test for unpaired samples were performed for two groups using Graph Pad Prism version 5 (Graph Pad Software, Inc., San Diego, CA, USA). p < 0.05 was considered as statistically significant.

## 3 Results

### 3.1 PD1 expression on CD4+CD25+FoxP3+ Tregs predominated in PBMCs of leprosy patients

**Figure 1:**
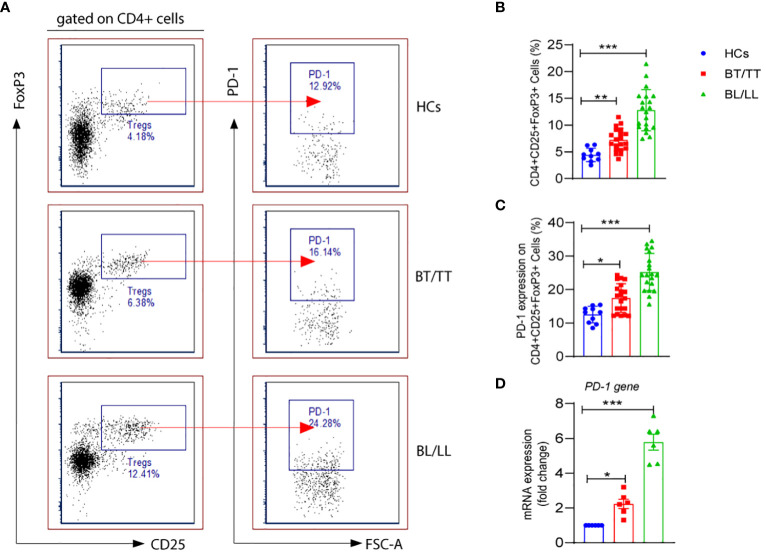
PD-1 expression on T regulatory cells in Leprosy **(A)** FACS plot showing the frequency of Treg (CD4+CD25+FoxP3) cells expressing programmed death 1 (PD-1) of one representative plot of each group is shown as determined by FACS analysis in healthy controls (n=10), tuberculoid patients (n=20) and lepromatous leprosy patients (n=20). **(B)** Scattered dot plots are showing expression of PD-1 on gated CD4+CD25+ FoxP3+ cells in different groups of Leprosy patients (BT/TT vs. BL/LL) and Healthy Contacts (n = 10) **(C)** Scattered dot plots are showing mRNA expression of PD-1 gene in isolated PBMCs from different groups of Leprosy patients (BT/TT vs. BL/LL, n=6) and Healthy Contacts (n = 6). Mean ± SD values are shown in each set while significance was determined by a P value of 0.05. A total of 100,000 cells were analyzed by flow cytometry. FCS express software was used for data analysis and Statistical analysis was done using one-way ANOVA for unpaired samples (*p < 0.05; **p < 0.005; ***p < 0.0005).

As CD4+ T lymphocytes, which are also referred to as helper cells, they play a crucial role in regulating the immune response by either activating other immune cells or dividing into Treg cells to suppress the immune response. In the present study, we first evaluated the percentages of CD4+CD25+FoxP3+ Tregs in the PBMCs of leprosy patients as well as healthy controls. The percentage of CD4+CD25+FoxP3+ Tregs was significantly higher in the PBMCs of lepromatous leprosy patients (12.53 ± 2.46%) compared with that in tuberculoid leprosy patients (7.15 ± 1.71%) and healthy individuals (4.42 ± 1.23%) (**Figures 1A, B**). PD-1 is one of the inhibitory molecules involved in immune suppression. We assessed the expression of PD-1 on CD4+CD25+FoxP3+ Tregs derived from leprosy patients and healthy individuals to determine if it contributes to immune suppression. The expression levels of PD-1 on Tregs were significantly higher in the PBMCs of lepromatous (25.11 ± 5.13%) and tuberculoid patients (17.49 ± 4.30%) as compared to healthy individuals (12.53 ± 2.46%) (**Figures 1A, C**). Moreover, we observed significant increase in the PD-1 gene expression at mRNA level in lepromatous as well tuberculoid leprosy patients (**Figure 1D**). In addition, we examined the expression of PDL-1 on T cells in PBMCs from a group of patients with leprosy. The frequency of CD3+PD-L1+ T cells among patients with leprosy was significantly higher than in healthy controls (**Supplementary Figure S1**). Taken together our results indicated that PD-1 expression on CD4+CD25+FoxP3+ cells increased in leprosy patients, it may contribute to immunosuppression of the host.

### 3.2 PD-1^neg^ Tregs from leprosy patients produce higher IL-10 than PD-1^+^ Tregs

**Figure 2:**
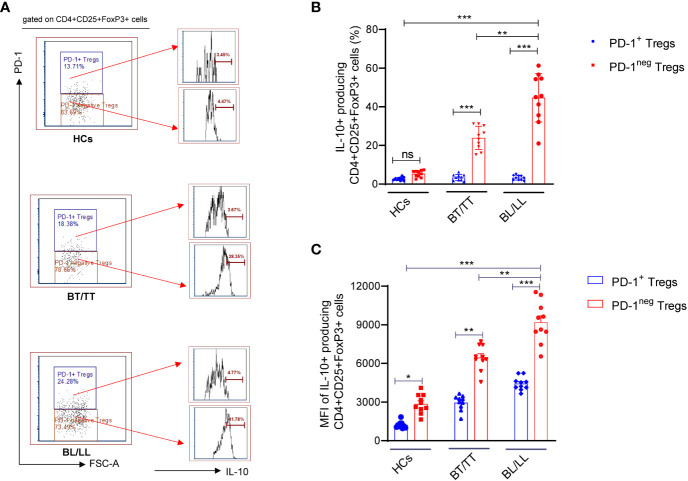
IL-10 production by PD-1^neg^ and PD-1+T regulatory cells in Leprosy **(A)** Histogram FACS cartoon showing one representative plot of each group IL-10 production by PD-1^neg^ and PD-1+ Tregs as determined by FACS analysis in healthy controls (n=10), tuberculoid patients (n=10) and lepromatous leprosy patients (n=10) **(B)** Scattered dot plots are showing the IL-10 production by PD-1^neg^ and PD-1+ Tregs in different groups of Leprosy patients (BT/TT vs. BL/LL) and Healthy Contacts (n = 10) **(C)** Scattered dot plots are showing the mean fluorescence intensity (MFI) of IL-10 producing by PD-1^neg^ and PD-1+ Tregs in different groups of Leprosy patients (BT/TT vs. BL/LL) and Healthy Contacts (n = 10). Mean ± SD values are shown in each set while significance was determined by a P value of 0.05. A total of 100,000 cells were analyzed by flow cytometry. FCS express software was used for data analysis and Statistical analysis was done using one-way ANOVA for unpaired samples (*p < 0.05; **p < 0.005; ***p < 0.0005).

It is important to understand that T-regulatory cells are immune suppressive cells, which exert their immunosuppressive effect through the production of factors such as IL-10, IL-35, and TGF-β. According to a study conducted on leprosy patients, FoxP3+ Treg cells have been shown to down-regulate T cell responses, which results in both the characteristic antigen-specific anergy in leprosy patients and the characteristic leprosy-induced symptoms of inflammation. Although, it remains to be investigated whether PD-1 plays a role in the Treg-mediated immunosuppression in leprosy patients and how it could contribute to this. With this intention, we investigated the effect of PD-1 expression on IL-10 production by Tregs in leprosy patients as IL-10 is one of the main immunosuppressive cytokines secreted by Tregs. We investigated the production of IL-10 by PD-1^neg^ and PD-1^+^ Treg cells in stimulated PBMC cultures of leprosy patients and healthy controls that were treated with *M. leprae* antigen. By flow cytometry, we determined the intracellular production of IL-10 by enriched PD-1^neg^ Tregs and PD-1+ Tregs in leprosy patients and healthy controls. The percentage of IL-10+ produced by PD-1^neg^ Treg was significantly higher among BL/LL patients (44.75 ± 12.46%) (**Figure 1D**) compared with BT/TT patients (23.94 ± 5.97%) and healthy controls (5.34 ± 1.38%) (**Figures 2A, B**). However, we have observed that PD-1+ Tregs are producing very little amount of IL-10 in all the patients group as well as healthy controls (**Figures 2A, B**). We also found significant increase mean fluorescence intensity (MFI) in BL/LL and BT/TT patients compared to healthy controls (**Figure 2C**). The increased frequency of IL-10-producing PD-1^neg^ Tregs in BT/TT and BL/LL patients, suggesting that they play a suppressive function in a contact-independent manner *via* the release of IL-10.

### 3.3 PD-1 mAb blocking enhances treg cell-mediated immunosuppression in leprosy patients

**Figure 3:**
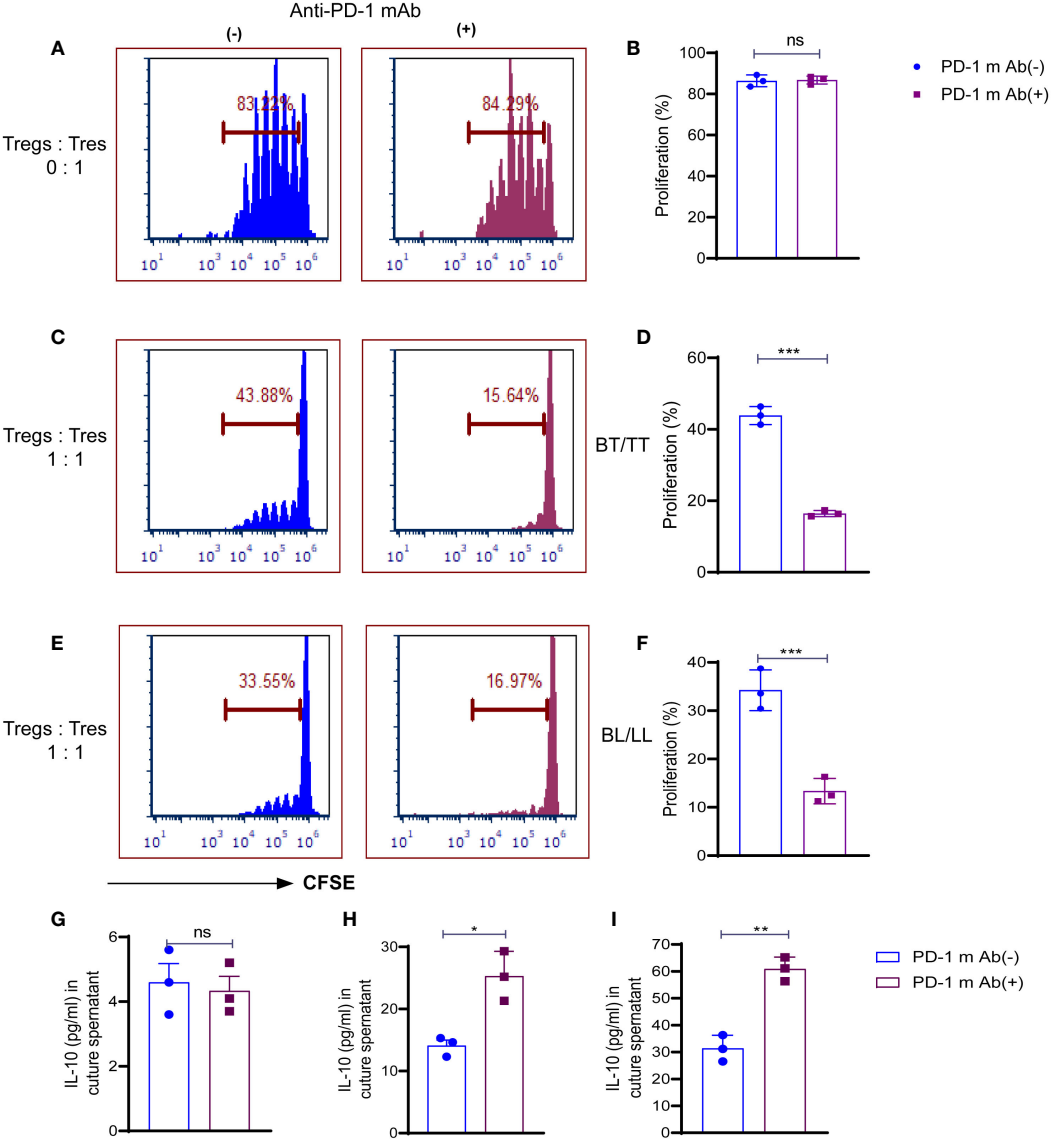
Role of PD-1 in Treg cell-mediated immunosuppression in leprosy. CSFE labelled CD8+T cells (Tresp cells) from PBMCs of healthy control cultured with irradiated APCs cultured and ant-CD3 with and without anti-mAb of PD-1. **(A)** Histogram showing percent of proliferating Tresp cells in the cultures with and without anti-mAb of PD-1 (15 µg/ml) with PD-1+Treg sorted cells from healthy controls (n=3) **(B)** Scattered dot plots showing proliferation of CD8+T cells (Tresp cells) without co-culture of Tregs, and CFSE-labeled CD8+T cells (Tresp cells) were cultured for 5 days with anti-CD3 mAb and irradiated APCs. Proliferation of Tresp cells was determined by CFSE dilution with and without PD-1 blocking (15 µg/ml). **(C)** Histogram **(D)** Scattered dot plots showing proliferation of CD8+T cells (Tresp cells) when PD-1+Treg sorted cells from BT/TT patients (n=3), and CFSE-labeled CD8+T cells (Tresp cells) were cocultured for 5 days with anti-CD3 mAb and irradiated APCs. Proliferation of Tresp cells was determined by CFSE dilution with and without PD-1 blocking (15 µg/ml). **(E)** Histogram **(F)** Scattered dot plots showing proliferation of CD8+T cells (Tresp cells) when PD-1+Treg sorted cells from BL/LL patients (n=3), and CFSE-labeled CD8+T cells (Tresp cells) were cocultured for 5 days with anti-CD3 mAb and irradiated APCs. Proliferation of Tresp cells was determined by CFSE dilution with and without PD-1 blocking (15 µg/ml). **(G)** IL-10 cytokine levels were estimated by ELISA in culture supernatant of co-culture with T responder and without Tregs **(H, I)** IL-10 cytokine levels were estimated by ELISA in culture supernatant of co-culture with T responder and Tregs derived from BT/TT and BL/LL leprosy patients respectively. FCS express software was used for data analysis and Statistical analysis was done using Student’s t test samples (*p < 0.05; **p < 0.005; ***p < 0.0005).

Treg cells maintenance and immunosuppressive function are directly influenced by both TCR and CD28 signals, which are inhibited by PD-1. We examined whether the PD-1 blockade could affect the ability of Treg cells to suppress the T responder immune cell. In the first step, we analyzed the proliferative capacity of CD8+ T cells labeled with carboxyfluorescein diacetate succinimidyl ester (CFSE) in the presence of anti-CD3 mAb and antigen-presenting cells (APCs), which were cultured with or without PD-1+ Treg cells derived from BT/TT and BL/LL patients. In the absence of PD1+ Treg cells, responding CD8+ T cells (T responder cells) were seen to proliferate vigorously with PD-1 blocking (84.29%) and without PD-1 blocking (83.22%) (**Figures 3A, B**). This result indicating that blocking on PD-1 does not have any effect on proliferation in the absence of Tregs. Co-culture of PD1+ Tregs derived from BT/TT patients and CFSE labelled CD8+ T cells (T responder cells) showed 42.53% proliferation (**Figures 3C, D**) while BL/LL patients derived PD1+ Treg showed 33.88% proliferation (**Figures 3E, F**). Further significant enhancement of suppression by PD1+ Treg cells was observed when anti-PD-1 mAb was added to the cell culture in both groups of leprosy patients (**Figures 3C–F**). The findings collectively suggest that PD-1 expressed by Treg cells in leprosy patients may act as a negative regulator of Treg-mediated immunosuppressive function and T cell proliferation, and also suggest that blocking PD-1 helps to enhance Treg cell proliferation and immunosuppressive activity. These results also indicating that BL/LL patients derived Tregs have more suppressive activity than Tregs of BT/TT leprosy patients and it may be IL-10 dependent.With this intend we estimated the IL-10 cytokine in the culture supernatant of co-culture of Tresponder and Trregs with and without blocking of PD-1. We observed no difference in the IL-10 level with and PD-1 blocking in case of Tresponder only (**Figure 3G**). However, there is significant difference in IL-10 level upon PD-1 blocking in both groups of leprosy patients (**Figures 3H, I**). These results collectively suggested that PD-1 blocking enhanced Treg suppressive activity in IL-10 dependent manner as we observed increased IL-10 levels in the co-culture supernatant upon blocking the PD-1.

### 3.4 Leprosy patients have increased expression of PD-1 and its ligand(s) on T cells, B cells, and monocytes

**Figure 4:**
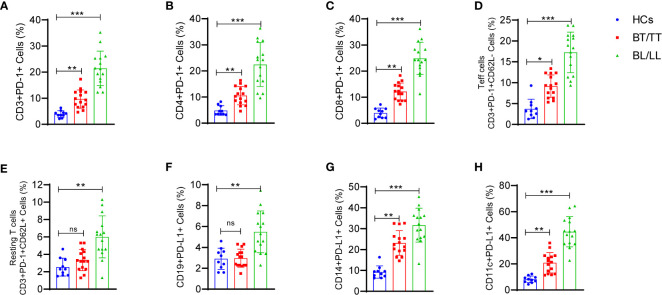
Increased frequency of T cell expressing programmed death 1 (PD-1) and its ligand (PD-L1) on CD14+, CD19+ and CD11c+ cells in Leprosy: Scattered dot plots are showing the expressing programmed death 1 (PD-1) on **(A)** T cells (CD3+PD-1+), **(B)** T helper cells (CD4+PD-1+), **(C)** T cytotoxic cells (CD8+PD-1+), **(D)** Effector T cells (CD3+PD-1+CD62L-), **(E)** Resting T cells (CD3+PD-1+CD62L+) and PD-L1 expression on **(F)** CD19+ B cells **(G)** CD14+ monocytes and **(H)** CD11c+ cells as determined by FACS analysis in healthy controls (n=10), tuberculoid patients (n=10) and lepromatous leprosy patients (n=10). Mean ± SD values are shown in each set while significance was determined by a P value of 0.05. A total of 100,000 cells were analyzed by flow cytometry. FCS express software was used for data analysis and Statistical analysis was done using one-way ANOVA for unpaired samples (*p < 0.05; **p < 0.005; ***p < 0.0005).

PD-1-mediated suppression of T-cell effector functions relies upon the engagement of its ligand with the antigen presenting cells (APCs) or T-cells that express its ligand. Thus, we determined PD-1 expression CD3+, CD4+, CD8+ T cells, activated (CD62L-) CD3+T cells, resting (CD62L+) and PD-L1 expression on CD19+ B, CD14+ monocytes, CD11c+ dendritic cells and how they react with the PD-1. There was a significant increase in PD-1-expressing CD3+ T cells in leprosy patients compared to healthy controls (**Figure 4A**). In addition, we examined whether PD-1 was expressed on CD4+ and CD8+ T cells in the peripheral blood of leprosy patients and found that their frequencies of PD-1-expressing CD4+ and CD8+ T cells were significantly higher compared to healthy controls (**Figures 4B, C**). In the next step, we analyzed PD-1 expression on CD3+T cells of patients with leprosy based on CD62L expression, on effector (CD62L-) CD3+T cells and resting (CD62L+) CD3+T cells of leprosy patients. PD-1 expression was primarily observed on activated T cells (**Figure 4D**), whereas resting T cells (**Figure 4E**) were significantly less expressed among leprosy patients. APCs or T-cells expressing PD-1 ligand are necessary for PD-1-mediated suppression of T-cell effector functions. Hence, we investigated the expression of PD-L1 on CD14+monocytes, CD11c+ dendritic cells and CD19+B cells to determine if PD-1 influences these cells. We observed that there are significant increases in the percentage of CD19+ expressing PD-L1 (**Figure 4F**), particularly among patients with leprosy. Additionally, we found that PD-L1 expression increased significantly on CD14+ monocyte (**Figure 4G**), and CD11c+ dendritic cells (**Figure 4H**) cells. The results suggest that *M. leprae* infection increases expression of PD-1 on T cells and its ligand(s), particularly PD-L1, on APCs which may contribute to T cell anergy in leprosy patients.

### 3.5 The relationship between PD-1 expressing cell subsets and bacterial index in leprosy patients

**Figure 5:**
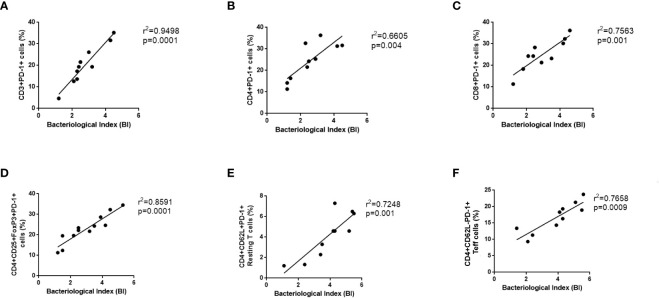
Positive Correlation between PD-1 expressing various T cells and bacteriological index (BI) in Leprosy. PD-1 expression on various T cells **(A)** CD3+PD-1+ **(B)** T helper cells (CD4+PD-1+) **(C)** T cytotoxic cells (CD8+PD-1+), **(D)** T regulatory cells (CD4+CD25+FoxP3+PD-1+) **(E)** T effector cells (CD3+PD-1+CD62L-), **(F)** Resting T cells (CD3+PD-1+CD62L+) positively correlates with bacteriological index (BI) in leprosy patients (n = 10). Correlation was done using Spearman’s rank correlation coefficient.

There has been a direct correlation between the PD-1 signaling axis and anergy in T-cells, pathogen persistence, and peripheral immune tolerance during the recent past but its correlation with leprosy is not fully explored. It is well known that the expression of PD-1 on a wide variety of T cells plays a crucial role in shaping the immune response of the host as well as the development of disease. Hence, we correlate the PD-1 expressing CD3, CD4, CD8 cells, resting T cells, activated T cells and Tregs with bacteriological index (BI) in leprosy patients (**Figures 5A–F**). Moreover, the proportion of BI also exhibited a positive correlation with an increased proportion of PD-L1(data not shown). These results suggested that the concomitant increase in BI in leprosy patients may inhibit T cell activity by resulting in anergy and exhaustion caused by high PD-1 expression on Tregs, helper and cytotoxic T cells.

## 4 Discussion

There is evidence that Treg cells suppress T-cell effectors (Teff cells) during chronic bacterial or viral infections to limit the damaging effects of immune responses against pathogens (25). The regulatory T (Tregs) in the peripheral blood play an important role in maintaining peripheral tolerance, preventing autoimmune diseases, and limiting chronic inflammation. The possibility of hyporesponsiveness of T cells in leprosy patients has been investigated in several studies as being caused by several factors, including foamy macrophages in the presence of IL-10 and Tregs. In several studies, Treg cells have been demonstrated to suppress immune responses in the periphery of leprosy patients and markers associated with Treg cells, mostly CD25, TGF-1, IL-35, CTLA4, IL-10, and FoxP3. The frequency of IL-10 producing Bregs also found high in leprosy patients, and it is involved in the pathogenesis of leprosy. The predominant sign of lepromatous multibacillary leprosy is reduced *M leprae* specific T-cell mediated immunity. This can be observed both *in vitro* (unresponsiveness to *M leprae* antigens) and clonally (clonal anergy) (26, 27). It has been found that the PD-1 signaling axis is highly related to the anergy of T-cells, pathogen persistence, and peripheral tolerance of immunity (28). PD-1 is a negative costimulatory molecule that exerts a negative effect on T cells by inhibiting cytokine production and cell proliferation by decreasing the expression of cytokines (28).

It has been demonstrated that cytokine production and proliferation by virus-specific CD4 and CD8 T cells can be enhanced or restored by blocking the PD-1 signaling pathway, either in animal studies or *in vivo* studies (29, 30). The overexpression of PD-1 T cells and the inhibitory function of these cells have been reported in patients with tuberculosis and inhibition PD-1/PD-L1 increased production of IFN-γ (31) and leprosy (22). In one study, blocking PD-1/PD-L1 signaling for 6 hours did not significantly increase the production of Mtb-specific IFN-γ (32). A definitive understanding of PD-1 and its ligand PD-L1, as well as its mechanism of inhibiting *M. leprae* specific T-cell responses, is still unclear. Our study found that PD-1 expressing CD4+CD25+FoxP3+ Treg cells were more prevalent in lepromatous leprosy and tuberculoid patients compared to healthy controls which is consistent with other studies. Similar trend was found in case of PDL-1 expression on Tregs. Furthermore, Treg cells produce significant amounts of IL-10 in leprosy patients (33), indicating that these cells are suppressive in nature. Next, we analyzed the effect of surface expression of PD-1 on IL-10 production by Treg. We found PD-1 expressing Tregs secreting very less amount of IL-10 compared to PD-1^neg^ Tregs which is secreting very amount of IL-10 in leprosy patients.

Efficacy of Treg cells can be attributed to the suppression of both contact-dependent and contact-independent effector T cells (34). There has been considerable attention paid to inhibitory cytokines such as IL10, TGF-b, IL-35, as mediators of Treg cell suppression (13, 35). PD-1 and IL-10 pathways are upregulated in chronic infections and cancers as a result of antigen persistence, impairing immunity and advancing disease (36). The function of PD-1-expressing Tregs is critical for leprosy immunopathogenesis. As a next step, we examined whether PD-1 on Tregs plays any role in the immune suppression. So, we were inclined to block PD-1 Tregs *in vitro* in PBMC cultures of leprosy patients and observe their suppression activity on effector T cells. The immunosuppressive activity of PD-1–deficient Treg cells was shown to be greater than that of PD-1–intact Treg cells, which indicates that PD-1 signaling enhances Treg cell immunosuppressive properties (37).

A PD-1 blockade on Tregs decreased effector T cell proliferation and increased suppressive activity of Tregs. As shown in a previous animal study, PD-1–deficient Treg cells had significantly increased immunosuppressive activity and improved protection against autoimmune diseases, suggesting that Treg cells with less PD-1 signaling have a stronger immunosuppressive function (37). In addition, Treg cells inhibit the immune response of CD8+ T cells by interacting directly with PD-1 on Treg cells (38). A dual blockade of IL-10 and PD-1 appears to be more effective in restoring antiviral CD8+ and CD4+ T cell responses and viral clearance in chronic viral infections than either single blockade alone. Both pathways act synergistically through distinct pathways to suppress T cell functions (39, 40). Our study confirmed that blocking of PD-1 increases suppressive activity of Tregs in leprosy patients in IL-10 dependent fashion.

It is a well-known fact that co-stimulatory and co-inhibitory molecules play important roles in controlling T cell responses to infections (41, 42). In the context of T cell function, the PD-1-PDL-1 interaction inhibits the function of the effector T cells, such as proliferation, cytokine production (43). The expression of PDL-1 on the APCs in leprosy leads to an ineffective antigen-specific immune response, leading to *M. leprae* persistence, as was demonstrated recently by our laboratory. We observed that the surface expression of PD-1 on CD3, CD4, CD8, resting and activated T cells is significantly higher in patients with BL/LL than in BT/TT patients or healthy individuals. There was a higher expression of PDL-1 on CD14+ monocytes, CD11c+ dendritic cells and CD19+ B cells, suggesting that these cells play a role in the impairment of the innate immune system in leprosy. As a result, this may be considered as one of the mechanisms by which Treg cells suppress the immune system in a contact-dependent manner. We also observed that high bacillary load (BI) and elevated PD-1 expression levels on various T cells have a positive correlation in lepromatous patients. To understand how bacillary load (BI) plays a role in the development and maintenance of this population, further studies in leprosy patients are required. Studies have shown that PD-1/PD-L1 suppress IFN-γ against TNF-α in leprosy patients. Treg cells may also utilize this technique for suppressing effector T cells *via* contact-dependent mechanisms (44).

It might be possible that high BI increased Tregs as well as PD-1 expression on other immune cells. It has been shown that PD-1 blockade leads to both the recovery of dysfunctional PD-1+CD8+ T cells as well as the enhancement of PD-1+ Treg-mediated immunosuppression in cancer. By blocking PD-1, tumor regression requires profound reactivation of effector PD-1+CD8+ T cells rather than PD-1+ Treg cells (45).

Taken together, our study suggested that PD-1 expression is increased on various T cells in leprosy patients, which may contribute to T cell anergy. PD-1 blockade of Tregs promotes Treg cell-mediated immune suppression of T effector cells. Thus, based on our studies, we are pointing out the importance of PD-1 expressing Tregs in leprosy pathogenesis, we have a better understanding of the immunological features that mediate and regulate the immune suppression in leprosy patients.

## Data availability statement

The raw data supporting the conclusions of this article will be made available by the authors, without undue reservation.

## Ethics statement

The studies involving human participants were reviewed and approved by Institute Ethics Committee, AIIMS in New Delhi, India (IESC/T-417/01.11.2013). Written informed consent for participation was not required for this study in accordance with the national legislation and the institutional requirements.

## Author contributions

Conceptualization, MT. methodology, MT. software, HN. validation, CS. formal analysis, AT, and NM. investigation, HS. resources, NK and AS. data curation, MT, and HS. writing—original draft preparation, MT. writing—review and editing, MT, MS, NK, HN and AS. visualization, CS. supervision, AS. project administration, AS. funding acquisition, MS. TZ & TK: Reanalyzed data and revised the figures and graphs. All authors contributed to the article and approved the submitted version.

## References

[1] Ridley DS Jopling WH. Classification of leprosy according to immunity. a five-group system. Int J Leprosy Other Mycobacterial Dis (1966) 34:255–73. 5950347

[2] Montoya D Modlin RL. Learning from leprosy: insight into the human innate immune response. Adv Immunol (2010) 105:1–24. doi: 10.1016/S0065-2776(10)05001-7 20510728

[3] Salgame P Abrams JS Clayberger C Goldstein H Convit J Modlin RL. Differing lymphokine profiles of functional subsets of human CD4 and CD8 T cell clones. Sci (New York N.Y.) (1991) 254:279–82. 10.1126/science.254.5029.279 1681588

[4] Yamamura M Uyemura K Deans RJ Weinberg K Rea TH Bloom BR. Defining protective responses to pathogens: cytokine profiles in leprosy lesions. Sci (New York N.Y.) (1991) 254:277–9. 10.1126/science.254.5029.277 1925582

[5] Saini C Tarique M Rai R Siddiqui A Khanna N Sharma A. T Helper cells in leprosy: An update. Immunol Lett (2017) 184:61–6. doi: 10.1016/j.imlet.2017.02.013 28235552

[6] Tarique M Saini C Naz H Naqvi RA Khan FI Sharma A. Fate of T cells and their secretory proteins during the progression of leprosy. Curr Protein Pept Sci (2018) 19:889–99. doi: 10.2174/1389203718666170829120729 28847289

[7] Kumar S Naqvi RA Khanna N Pathak P Rao DN. Th3 immune responses in the progression of leprosy *via* molecular cross-talks of TGF-β, CTLA-4 and cbl-b. Clin Immunol (Orlando Fla.) (2011) 141:133–42. 10.1016/j.clim.2011.06.007 21807564

[8] Tarique M Saini C Naqvi RA Khanna N Rao DN. Increased IL-35 producing tregs and CD19+IL-35+ cells are associated with disease progression in leprosy patients. Cytokine (2017) 91:82–8. doi: 10.1016/j.cyto.2016.12.011 28038394

[9] Tarique M Saini C Naqvi RA Khanna N Sharma A Rao DN. IL-12 and IL-23 modulate plasticity of FoxP3+ regulatory T cells in human leprosy. Mol Immunol (2017) 83:72–81. doi: 10.1016/j.molimm.2017.01.008 28110210

[10] Tarique M Naqvi RA Ali R Khanna N Rao DN. CD4+ TCRγδ+ FoxP3+ cells: An unidentified population of immunosuppressive cells towards disease progression leprosy patients. Exp Dermatol (2017) 26:946–8. doi: 10.1111/exd.13302 28109171

[11] Saini C Tarique M Ramesh V Khanna N Sharma A. γδ T cells are associated with inflammation and immunopathogenesis of leprosy reactions. Immunol Lett (2018) 200:55–65. doi: 10.1016/j.imlet.2018.07.005 30006101

[12] Sadhu S Khaitan BK Joshi B Sengupta U Nautiyal AK Mitra DK. Reciprocity between regulatory T cells and Th17 cells: Relevance to polarized immunity in leprosy. PloS Negl Trop Dis (2016) 10:e0004338. doi: 10.1371/journal.pntd.0004338 26751584 PMC4709061

[13] Saini C Ramesh V Nath I. Increase in TGF-β secreting CD4^+^CD25^+^ FOXP3^+^ T regulatory cells in anergic lepromatous leprosy patients. PloS Negl Trop Dis (2014) 8:e2639. doi: 10.1371/journal.pntd.0002639 24454972 PMC3894184

[14] Tarique M Naz H Kurra SV Saini C Naqvi RA Rai R. Interleukin-10 producing regulatory b cells transformed CD4+CD25- into tregs and enhanced regulatory T cells function in human leprosy. Front Immunol (2018) 9:1636. doi: 10.3389/fimmu.2018.01636 30083152 PMC6065098

[15] Saini C Srivastava RK Tarique M Kurra S Khanna N Ramesh V. Elevated IL-6R on CD4+ T cells promotes IL-6 driven Th17 cell responses in patients with T1R leprosy reactions. Sci Rep (2020) 10:15143. doi: 10.1038/s41598-020-72148-7 32934336 PMC7493991

[16] Belkaid Y Rouse BT. Natural regulatory T cells in infectious disease. Nat Immunol (2005) 6:353–60. doi: 10.1038/ni1181 15785761

[17] Belkaid Y. Regulatory T cells and infection: a dangerous necessity. Nat Rev Immunol (2007) 7:875–88. doi: 10.1038/nri2189 17948021

[18] Tarique M Naz H Saini C Suhail M Shankar H Khanna N. Association of IL-10 gene polymorphism with IL-10 secretion by CD4 and T regulatory cells in human leprosy. Front Immunol (2020) 11:1974. doi: 10.3389/fimmu.2020.01974 32849660 PMC7424005

[19] Chaves AT Ribeiro-Junior AF Lyon S Medeiros NI Cassirer-Costa F Paula KS. Regulatory T cells: Friends or foe in human mycobacterium leprae infection? Immunobiology (2018) 223:397–404. doi: 10.1016/j.imbio.2017.11.002 29150026

[20] Francisco LM Sage PT Sharpe AH. The PD-1 pathway in tolerance and autoimmunity. Immunol Rev (2010) 236:219–42. doi: 10.1111/j.1600-065X.2010.00923.x PMC2919275 20636820

[21] Virgin HW Wherry EJ Ahmed R. Redefining chronic viral infection. Cell (2009) 138:30–50. doi: 10.1016/j.cell.2009.06.036 19596234

[22] Sadhu S Kumar S Mitra DK Joshi B. Activated TLR2/4-positive T cells boost cell exhaustion during lepromatous leprosy infection *via* PD-1 upregulation. Heliyon (2022) 8:e11633. doi: 10.1016/j.heliyon.2022.e11633 36419668 PMC9676533

[23] Mi Z Wang Z Xue X Liu T Wang C Sun L. The immune-suppressive landscape in lepromatous leprosy revealed by single-cell RNA sequencing. Cell Discovery (2022) 8:2. doi: 10.1038/s41421-021-00353-3 35013182 PMC8748782

[24] Lima HR Gasparoto TH De Souza Malaspina TS Marques VR Vicente MJ Marcos EC. Immune checkpoints in leprosy: Immunotherapy as a feasible approach to control disease progression. Front Immunol (2017) 8:1724. doi: 10.3389/fimmu.2017.01724 29312289 PMC5732247

[25] Cools N Ponsaerts P Van Tendeloo VFI Berneman ZN. Regulatory T cells and human disease. Clin Dev Immunol (2007) 2007:89195. doi: 10.1155/2007/89195 18317534 PMC2253668

[26] Ottenhoff TH Elferink DG Klatser PR De Vries RR. Cloned suppressor T cells from a lepromatous leprosy patient suppress mycobacterium leprae reactive helper T cells. Nature (1986) 322:462–4. doi: 10.1038/322462a0 2426597

[27] Palermo ML Pagliari C Trindade M Yamashitafuji TM Duarte AJS Cacere CR. Increased expression of regulatory T cells and down-regulatory molecules in lepromatous leprosy. Am J Trop Med Hygiene (2012) 86:878–83. doi: 10.4269/ajtmh.2012.12-0088 PMC3335697 22556091

[28] Wu JM Young ML Wang TR Lin SJ Chang JK Wei J. Unusual cardiac malformations in Holt-oram syndrome: report of two cases. Zhonghua Minguo Xiao Er Ke Yi Xue Hui Za Zhi (1991) 32:100–4. 2063682

[29] Barber DL Wherry EJ Masopust D Zhu B Allison JP Sharpe AH. Restoring function in exhausted CD8 T cells during chronic viral infection. Nature (2006) 439:682–7. doi: 10.1038/nature04444 16382236

[30] Velu V Titanji K Zhu B Husain S Pladevega A Lai L. Enhancing SIV-specific immunity *in vivo* by PD-1 blockade. Nature (2009) 458:206–10. doi: 10.1038/nature07662 PMC2753387 19078956

[31] Singh A Mohan A Dey AB Mitra DK. Inhibiting the programmed death 1 pathway rescues mycobacterium tuberculosis-specific interferon γ-producing T cells from apoptosis in patients with pulmonary tuberculosis. J Infect Dis (2013) 208:603–15. doi: 10.1093/infdis/jit206 23661793

[32] Shen L Gao Y Liu Y Zhang B Liu Q Wu J. PD-1/PD-L pathway inhibits m.tb-specific CD4+ T-cell functions and phagocytosis of macrophages in active tuberculosis. Sci Rep (2016) 6:38362. doi: 10.1038/srep38362 27924827 PMC5141449

[33] Tarique M Naqvi RA Santosh KV Kamal VK Khanna N Rao DN. Association of TNF-α-(308(GG)), IL-10(-819(TT)), IL-10(-1082(GG)) and IL-1R1(+1970(CC)) genotypes with the susceptibility and progression of leprosy in north Indian population. Cytokine (2015) 73:61–5. doi: 10.1016/j.cyto.2015.01.014 25697140

[34] Hawrylowicz CM O'garra A. Potential role of interleukin-10-secreting regulatory T cells in allergy and asthma. Nat Rev Immunol (2005) 5:271–83. doi: 10.1038/nri1589 15775993

[35] Annacker O Asseman C Read S Powrie F. Interleukin-10 in the regulation of T cell-induced colitis. J Autoimmun (2003) 20:277–9. doi: 10.1016/S0896-8411(03)00045-3 12791312

[36] Porichis F Hart MG Zupkosky J Barblu L Kwon DS Mcmullen A. Differential impact of PD-1 and/or interleukin-10 blockade on HIV-1-specific CD4 T cell and antigen-presenting cell functions. J Virol (2014) 88:2508–18. doi: 10.1128/JVI.02034-13 PMC3958087 24352453

[37] Zhang B Chikuma S Hori S Fagarasan S Honjo T. Nonoverlapping roles of PD-1 and FoxP3 in maintaining immune tolerance in a novel autoimmune pancreatitis mouse model. Proc Natl Acad Sci United States America (2016) 113:8490–5. doi: 10.1073/pnas.1608873113 PMC4968716 27410049

[38] Stathopoulou C Gangaplara A Mallett G Flomerfelt FA Liniany LP Knight D. PD-1 inhibitory receptor downregulates asparaginyl endopeptidase and maintains Foxp3 transcription factor stability in induced regulatory T cells. Immunity (2018) 49:247–263.e247. doi: 10.1016/j.immuni.2018.05.006 30054205 PMC6105434

[39] Trautmann L Janbazian L Chomont N Said EA Gimmig S Bessette B. Upregulation of PD-1 expression on HIV-specific CD8+ T cells leads to reversible immune dysfunction. Nat Med (2006) 12:1198–202. doi: 10.1038/nm1482 16917489

[40] Brooks DG Ha S-J Elsaesser H Sharpe AH Freeman GJ Oldstone MBA. IL-10 and PD-L1 operate through distinct pathways to suppress T-cell activity during persistent viral infection. Proc Natl Acad Sci United States America (2008) 105:20428–33. doi: 10.1073/pnas.0811139106 PMC2629263 19075244

[41] Urbani S Amadei B Tola D Massari M Schivazappa S Missale G. PD-1 expression in acute hepatitis c virus (HCV) infection is associated with HCV-specific CD8 exhaustion. J Virol (2006) 80:11398–403. doi: 10.1128/JVI.01177-06 PMC1642188 16956940

[42] Keir ME Freeman GJ Sharpe AH. PD-1 regulates self-reactive CD8+ T cell responses to antigen in lymph nodes and tissues. J Immunol (Baltimore Md.: 1950) (2007) 179:5064–70. 10.4049/jimmunol.179.8.5064 17911591

[43] Jurado JO Alvarez IB Pasquinelli V Martínez GJ Quiroga MF Abbate E. Programmed death (PD)-1:PD-ligand 1/PD-ligand 2 pathway inhibits T cell effector functions during human tuberculosis. J Immunol (Baltimore Md.: 1950) (2008) 181:116–25. doi: 10.4049/jimmunol.181.1.116 18566376

[44] Sadhu S Mitra DK. Emerging concepts of adaptive immunity in leprosy. Front Immunol (2018) 9:604. doi: 10.3389/fimmu.2018.00604 29686668 PMC5900054

[45] Kumagai S Togashi Y Kamada T Sugiyama E Nishinakamura H Takeuchi Y. The PD-1 expression balance between effector and regulatory T cells predicts the clinical efficacy of PD-1 blockade therapies. Nat Immunol (2020) 21:1346–58. doi: 10.1038/s41590-020-0769-3 32868929

