# Supplementary figures and images for: Differential expression of programmed death 1 (PD-1) on various immune cells and its role in human leprosy
Source: Front Immunol. 2023 Apr 21;14:1138145. doi: 10.3389/fimmu.2023.1138145 (PMC10161389; doi:10.3389/fimmu.2023.1138145)

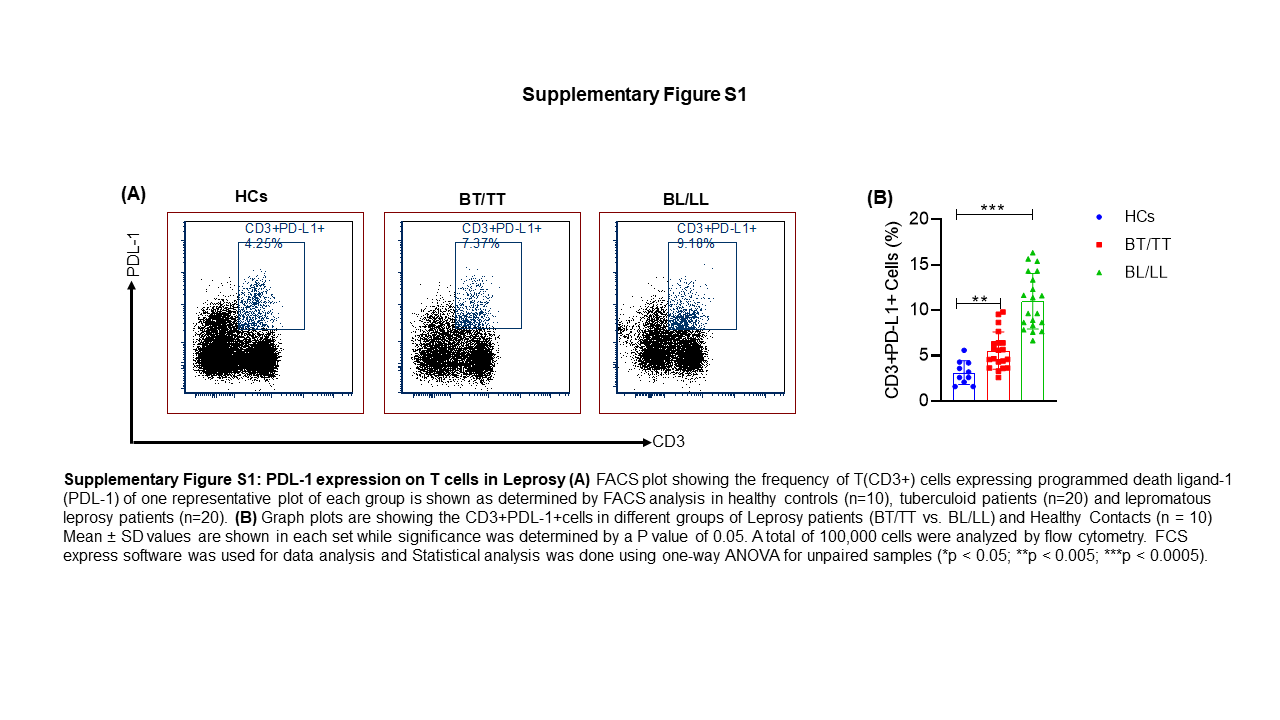

Supplement: Supplementary file 1 [file Image_1.tif]

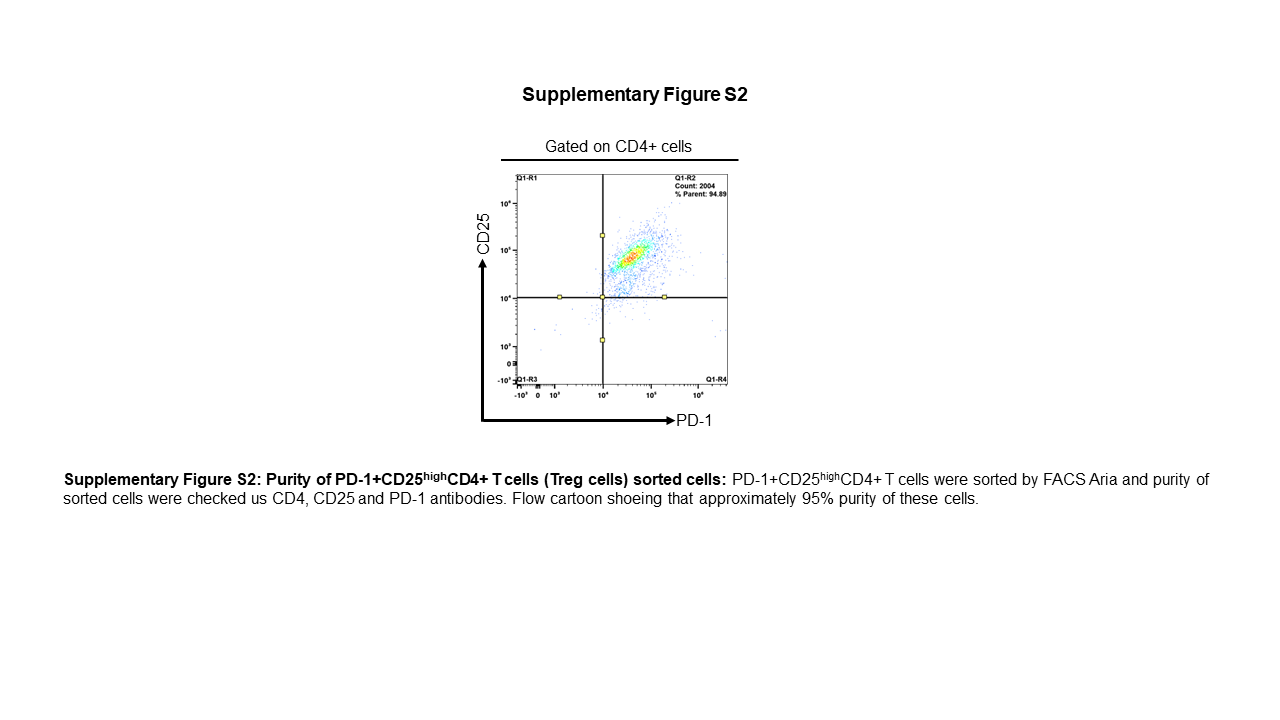

Supplement: Supplementary file 2 [file Image_2.tif]
